# Toll-Like Receptor 4 Mediates the Response of Epithelial and Stromal Cells to Lipopolysaccharide in the Endometrium

**DOI:** 10.1371/journal.pone.0012906

**Published:** 2010-09-22

**Authors:** Iain Martin Sheldon, Mark H. Roberts

**Affiliations:** Institute of Life Science, School of Medicine, Swansea University, Singleton Park, Swansea, United Kingdom; Universidad de la Republica, Uruguay

## Abstract

**Background:**

Ascending infections of the female genital tract with bacteria causes pelvic inflammatory disease (PID), preterm labour and infertility. Lipopolysaccharide (LPS) is the main component of the cell wall of Gram-negative bacteria. Innate immunity relies on the detection of LPS by Toll-like receptor 4 (TLR4) on host cells. Binding of LPS to TLR4 on immune cells stimulates secretion of pro-inflammatory cytokines such as IL-6, chemokines such as CXCL1 and CCL20, and prostaglandin E_2_. The present study tested the hypothesis that TLR4 on endometrial epithelial and stromal cells is essential for the innate immune response to LPS in the female genital tract.

**Methodology/Principal Findings:**

Wild type (WT) mice expressed TLR4 in the endometrium. Intrauterine infusion of purified LPS caused pelvic inflammatory disease, with accumulation of granulocytes throughout the endometrium of WT but not Tlr4^−/−^ mice. Intra-peritoneal infusion of LPS did not cause PID in WT or Tlr4^−/−^ mice, indicating the importance of TLR4 in the endometrium for the detection of LPS in the female genital tract. Stromal and epithelial cells isolated from the endometrium of WT but not Tlr4^−/−^ mice, secreted IL-6, CXCL1, CCL20 and prostaglandin E_2_ in response to LPS, in a concentration and time dependent manner. Co-culture of combinations of stromal and epithelial cells from WT and Tlr4^−/−^ mice provided little evidence of stromal-epithelial interactions in the response to LPS.

**Conclusions/Significance:**

The innate immune response to LPS in the female genital tract is dependent on TLR4 on the epithelial and stromal cells of the endometrium.

## Introduction

Ascending infection of the upper female genital tract with bacteria causes pelvic inflammatory disease (PID) in women, with an influx of granulocytes such as neutrophils and macrophages into the endometrium [1]. In the USA, about 1 million women seek treatment for acute PID each year with healthcare costs of $4 billion per annum [2]. Infection of the female genital tract with bacteria is also an important cause of preterm labour [3]. Intrauterine infusion of bacteria or their pathogen-associated molecules (PAMPs) such as lipopolysaccharide, have been used in mice to model PID [4], and in pregnant mice to mimic preterm labour [5]. Lipopolysaccharide (LPS) is the major structural component of the cell wall of Gram-negative bacteria but is also the endotoxin responsible for much of the inflammation and shock associated with bacterial infection [6].

Innate immunity in mammals is dependent on the detection of PAMPs by pattern recognition receptors such as the Toll-like Receptors (TLRs), and Toll-like receptor 4 was the first functional TLR to be discovered [7]. A receptor complex comprising of Toll-like receptor 4 (TLR4), CD14 and MD-2 on the cell membrane of host immune cells binds LPS [8]. Binding of LPS to TLR4 activates cell signalling pathways leading to an inflammatory response [9]. Typical inflammatory responses to LPS include secretion of the cytokines, interleukin (IL)-1β, IL-6, and tumour necrosis factor alpha (TNFα); the chemokines, chemokine (C-X-C motif) ligand 1 (CXCL1; also known as Keratinocyte-derived Cytokine, KC) and chemokine (C-C motif) ligand 20 (CCL20; also known as macrophage inflammatory protein 3 α, MIP-3α); and, prostaglandin E_2_ (PGE) [9]–[11].

Although most mucosa such as the alimentary and respiratory tracts have well organised aggregates of lymphoid tissue, the female genital tract does not, so innate immunity is particularly important for the defence of the endometrium [12]. Expression of TLRs is not limited to professional immune cells and endometrial cells express mRNA for the TLR4 receptor complex [13]–[15]. In addition, LPS stimulates the secretion of inflammatory mediators from endometrial cells [14]–[17]. However, it remains unclear whether TLR4 on endometrial cells is essential for the response to LPS in the female genital tract.

The present study tested the hypothesis that TLR4 on epithelial and stromal cells is essential for the innate immune response to LPS in the female genital tract. The Tlr4-deficient (Tlr4^−/−^) mouse [18], was used to examine the role of TLR4 on endometrial cells. Intrauterine infusion of purified LPS caused PID in wild type (WT) but not Tlr4^−/−^ mice *in vivo*. Furthermore, stromal and epithelial cells isolated from the endometrium of WT but not Tlr4^−/−^ mice, secreted inflammatory mediators in response to LPS *in vitro*. So, TLR4 is essential for the detection of LPS in the endometrium, with epithelial and stromal cells having a key role in innate immunity.

## Results

### TLR4 is necessary for the detection of LPS in the endometrium *in vivo*

**Figure 1:**
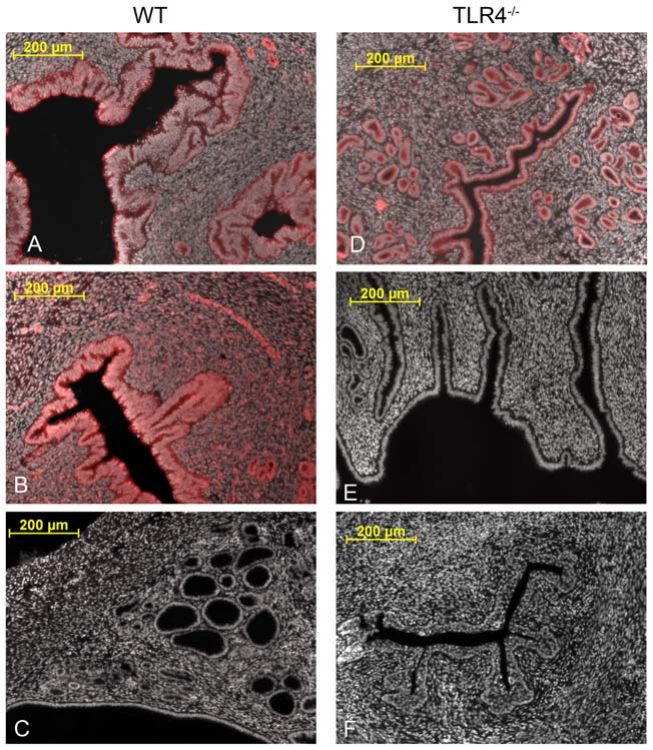
TLR4 is evident in the endometrium of wild type but not Tlr4^−/−^ mice. Representative photomicrographs of cross sections of uteri collected from wild type (WT; A–C; n = 6) or Tlr4^−/−^ mice (D–F; n = 6) immunostained (red) for pan cytokeratin (A, D), TLR4 (B, E), or with isotype control serum (C, F), and cell nuclei counterstained with DAPI (white). Four sections per animal were examined and at least 4 fields per section, with images collected at 10X magnification (bars  = 200 µm). Only WT mice showed evidence of TLR4 in the endometrium (B).

To examine if endometrial cells expressed TLR4, cross-sections of uteri from C57BL/6 wild type (WT) mice and Tlr4-deficient (Tlr4^−/−^) mice on the C57BL/6 genetic background [18], were compared using immunohistochemistry (Fig. 1; n = 6 animals per genotype with 4 uterine sections per animal). Using a pan cytokeratin antibody to highlight the epithelial cells, the architecture of the endometrium appeared similar between WT and Tlr4^−/−^ mice (Fig. 1A, D). The stromal and epithelial compartments of the endometrium of WT but not Tlr4^−/−^ mice had immuno-reactive TLR4 (Fig. 1B,E).

**Figure 2:**
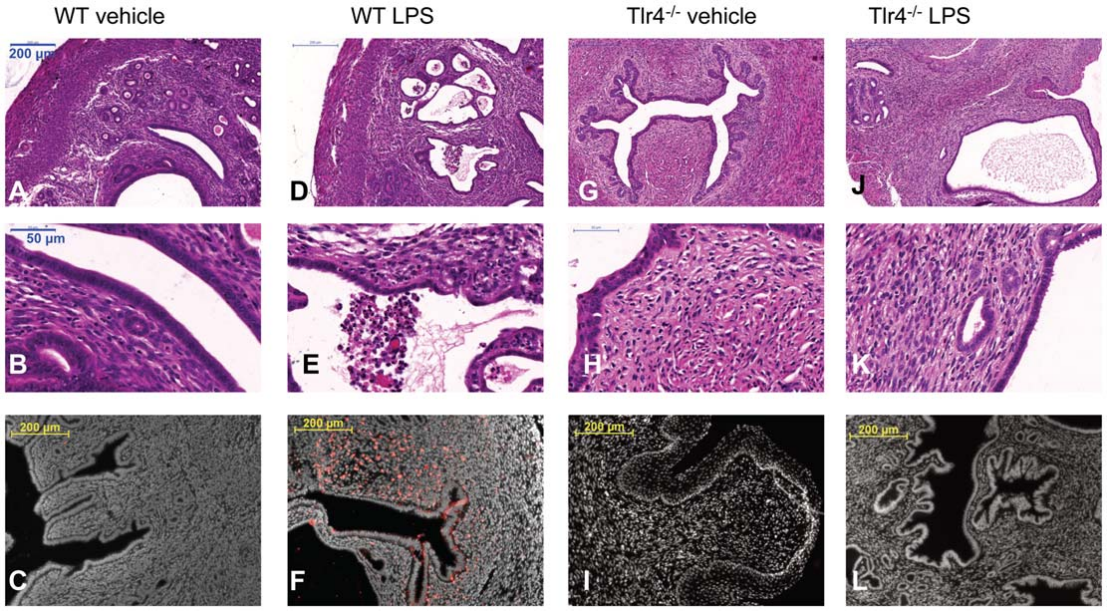
Intrauterine LPS stimulates inflammation in the endometrium of wild type but not Tlr4^−/−^ mice. Representative photomicrographs of cross sections of uteri collected from wild type (WT) mice 24 h after intrauterine infusion with 100 µl of vehicle (A–C; n = 14) or 100 µg ultrapure LPS from *E. coli* O111:B4 (D–F; n = 17), and Tlr4^−/−^ mice similarly infused intrauterine with vehicle (G–I; n = 8) or LPS (J–L; n = 8). Cross sections were stained with haematoxylin and eosin, and images collected at 10X magnification (A, D, G, J; bars  = 200 µm) or 40X magnification (B, E, H, K; bars  = 50 µm); or immunostained using a CD11b antibody for granulocytes (red) and DAPI for cell nuclei (white), and images collected at 10X magnification sections (C, F, I, L; bars  = 200 µm). At least 4 sections per animal and at least 4 fields per section were examined. Only WT mice infused with LPS show evidence of inflammation and accumulation of granulocytes in the endometrium (D–F).

**Figure 3:**
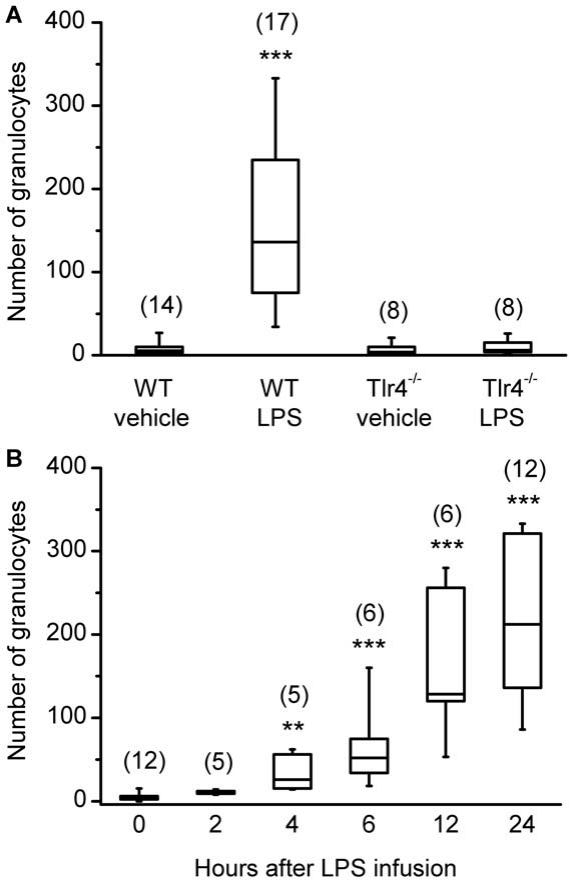
Intrauterine LPS stimulates accumulation of granulocytes in the endometrium of wild type but not Tlr4^−/−^ mice. Mice were infused intrauterine with 100 µl of vehicle or 100 µg ultrapure LPS from *E. coli* O111:B4, the uteri collected, cross sections immunostained using a CD11b antibody for granulocytes, and the number of CD11b immuno-reactive cells counted in 4 random areas averaged over 4 independent sections from each animal. (A) Boxplot of the number of granulocytes in the endometrium 24 h after wild type (WT) or Tlr4^−/−^ mice were infused intrauterine with vehicle or LPS. Only the WT mice infused with LPS had more granulocytes in the endometrium than WT mice infused with vehicle; data for each treatment group were compared with WT mice infused with vehicle using the Mann-Whitney *U* test, *** P<0.001. (B) Boxplot of the number of granulocytes in the endometrium 0, 2, 4, 6, 12 or 24 h after WT mice were infused with LPS. There was increasing accumulation of granulocytes in the endometrium from 4 to 24 h; data for each time point were compared with 0 h using the Mann-Whitney *U* test, ** P<0.01, *** P<0.001. In each boxplot, the box indicates the central range between the upper and lower quartile values, the solid horizontal bar represents the median, and the whiskers indicate the 95% central range; the numbers of animals are indicated in parentheses.

To examine if TLR4 is necessary for the detection of LPS in the endometrium, WT and Tlr4^−/−^ mice were infused intrauterine with 100 µl of vehicle or 100 µg ultrapure LPS from *E. coli* O111:B4. The uterus of WT mice appeared normal 24 h after infusion with vehicle (Fig. 2A, B; n = 14) and had few granulocytes as determined by immunohistochemistry using a CD11b antibody (Fig. 2C). However, WT mice infused intrauterine with LPS developed PID with histological evidence of inflammation (Fig. 2D, E; n = 17) and many granulocytes throughout the endometrium after 24 h (Fig. 2F). The Tlr4^−/−^ mice infused with vehicle (Fig. 2G–I; n = 8) or LPS (Fig. 2J–L; n = 8) did not show evidence of inflammation. These observations were confirmed by counting the number of CD11b immuno-reactive cells, a marker of granulocytes, in 4 random areas averaged over 4 independent cross-sections of the uterus from each animal. Wild type mice had more CD11b immuno-reactive cells when infused with LPS than vehicle (Fig. 3A). The number of cells did not differ significantly between the WT mice infused with vehicle and the Tlr4^−/−^ mice infused with vehicle or LPS (Fig. 3A). The number of granulocytes in the endometrium of WT mice increased with time after LPS infusion (Kruskal-Wallis test, P<0.001), with the first significant increase in the number of granulocytes 4 h after infusion (Fig. 3B).

**Figure 4:**
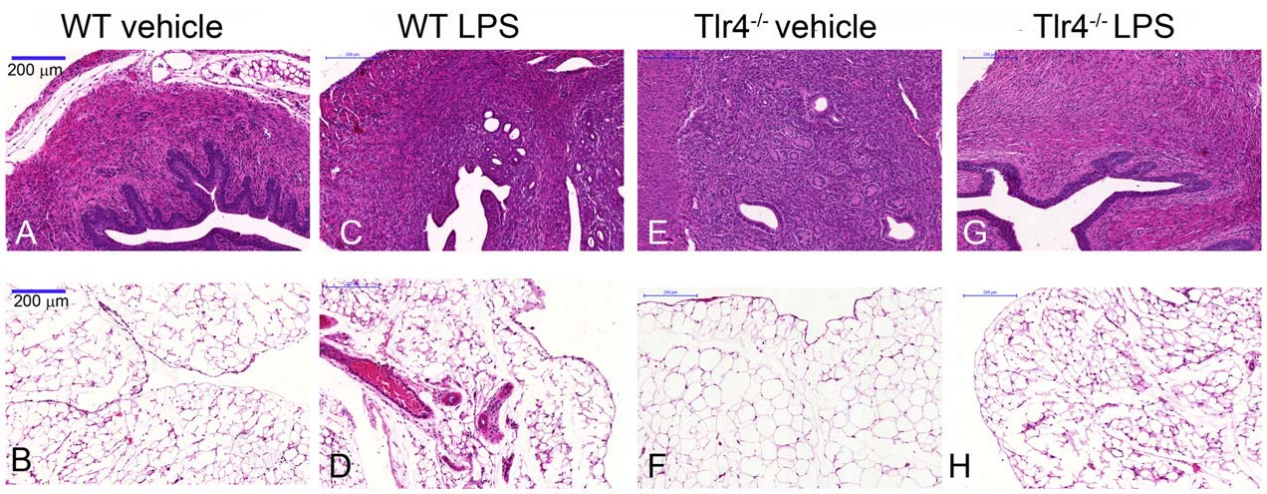
Intraperitoneal LPS does not stimulate inflammation in the endometrium. Representative photomicrographs of cross sections of uteri collected from wild type (WT) mice 24 h after intraperitoneal infusion with 100 µl of vehicle (A, B) or 100 µg ultrapure LPS from *E. coli* O111:B4 (C, D), and Tlr4^−/−^ mice similarly infused intraperitoneal with 100 µl vehicle (E, F) or 100 µg LPS (G, H). Cross sections of the uterus (A, C, E, G) or the peritoneal mesentery adjacent to the uterus (B, D, F, H) were stained with haematoxylin and eosin, and images collected at 10X magnification (bars  = 200 µm). At least 4 sections per animal and at least 4 fields per section were examined. There was no evidence of inflammation of the endometrium. However, WT mice treated with LPS had evidence of inflammation of the peritoneal mesentery with hyperaemia, extravasations of red blood cells and accumulation of granulocytes (D).

To confirm that the PID in WT mice treated with LPS was not a reflection of a generalised inflammatory response to LPS, the WT and Tlr4^−/−^ mice were also infused intra-peritoneal with 100 µl vehicle or 100 µg ultrapure LPS (n≥5 per group). The WT mice infused with vehicle or LPS did not develop PID (Fig. 4A–C), although there was inflammation of the peritoneal mesentery attached to the uterus of WT mice infused with LPS, with gross evidence of hyperaemia and a dull peritoneal surface as well as histological features of peritonitis (Fig. 4D). The Tlr4^−/−^ mice did not develop PID or peritonitis when infused intra-peritoneal with vehicle or LPS (Fig. 4E–H). The observations that TLR4 is expressed in the endometrium and intrauterine infusion of LPS causes PID, support the concept that the endometrial epithelial or stromal cells could be important for the localised detection and response to LPS.

### Endometrial stromal and epithelial cells respond to LPS *in vitro*

**Figure 5:**
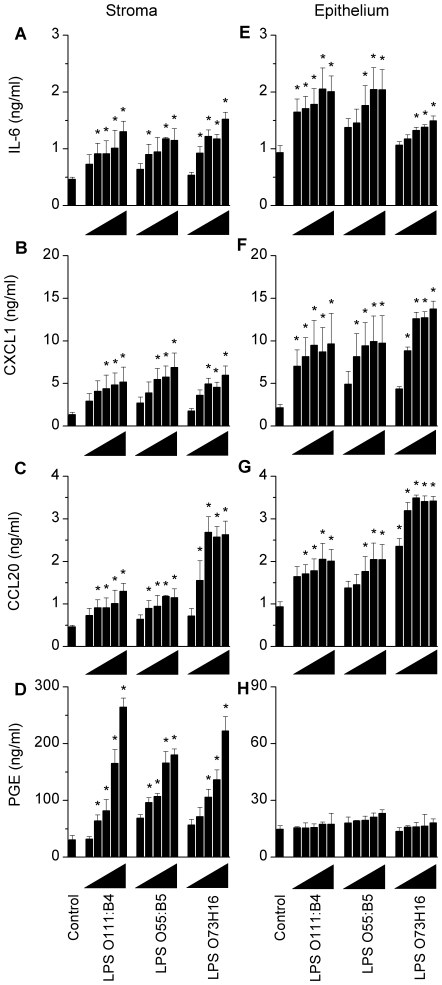
Endometrial cells secrete inflammatory mediators in response to LPS. Stromal (A–D) and epithelial cells (E–H) isolated from the endometrium of WT mice, were cultured for 24 h in medium (Control), or media containing LPS purified from *E. coli* serotype O111:B4, O55:B4, or O73:H16, at concentrations of 1, 10, 100, 1000, or 10,000 ng/ml LPS (represented by triangles). Supernatants were harvested to measure the accumulation of IL-6 (A, E), CXCL1 (B, F), CCL20 (C, G), and prostaglandin E_2_ (PGE; D, H). Each LPS stimulated accumulation of IL-6, CXCL1, CCL20, and PGE from stromal cells, and IL-6, CXCL1, and CCL20 from epithelial cells. Data are presented as mean + SEM and represent 3 independent experiments with 2 wells per treatment. Data were analysed by ANOVA, using the Dunnett's pairwise multiple comparison t-test to compare treatments with control, * P<0.05.

Immune responses to bacteria usually involve the secretion of pro-inflammatory cytokines, chemokines, and prostaglandins. In mice, typical responses to LPS include the secretion of cytokines such as IL-1β, IL-6 and TNFα; chemokines, including CXCL1 and CCL20; and, prostaglandin E_2_ (PGE) [9]–[11]. To examine if endometrial cells respond to LPS, stromal and epithelial cells were isolated from the endometrium of WT mice and treated for 24 h with LPS, using a range of concentrations on a ten-fold increasing scale from 1 ng/ml to 10 µg/ml, and the concentration of inflammatory mediators measured in supernatants. The structure of LPS can differ between strains of bacteria with consequences for the host response [19]. So, the present study examined three sources of LPS: ultrapure LPS from *E. coli* serotype O111:B4 (Invivogen); LPS purified from *E. coli* O55:B5 (Sigma); and, LPS purified from endometrial pathogenic *E. coli* serotype O73:H16, which was isolated from the endometrium of cattle with PID [4]. The supernatants from stromal cells accumulated IL-6, CXCL1, CCL20, and PGE in a concentration dependent manner for each LPS examined (Fig. 5A–D; ANOVA, P<0.05). The supernatants from epithelial cells also accumulated IL-6, CXCL1, and CCL20, in a concentration dependent manner for each LPS examined (Fig. 5E–G; ANOVA, P<0.05), although the epithelial cells secreted little PGE (Fig. 5H). The concentrations of IL-1β in the supernatant of cells treated with LPS were below the limits of detection of the assay (<12 pg/ml), and the concentrations of TNFα were close to or below the limits of detection of the assays (control vs. 1 µg/ml LPS; stroma: 27±3 vs. 72±12 pg/ml; epithelium: <15 vs. 42±7 pg/ml; Student's t-test, P<0.05). Stromal or epithelial cell survival did not differ significantly between control and LPS treatment (96.2±6.2 percent of control). Most endometrial cell inflammatory responses to the different sources of LPS were similar, although the LPS from *E. coli* serotype O73:H16 increased CCL20 production from stromal and epithelial cells compared with the other sources of LPS (Fig. 5C,G; Dunnett's pairwise multiple comparison t test, P<0.05). Subsequent experiments used ultrapure LPS from *E. coli* and focused on the accumulation of IL-6, CXCL1, CCL20, and PGE.

**Figure 6:**
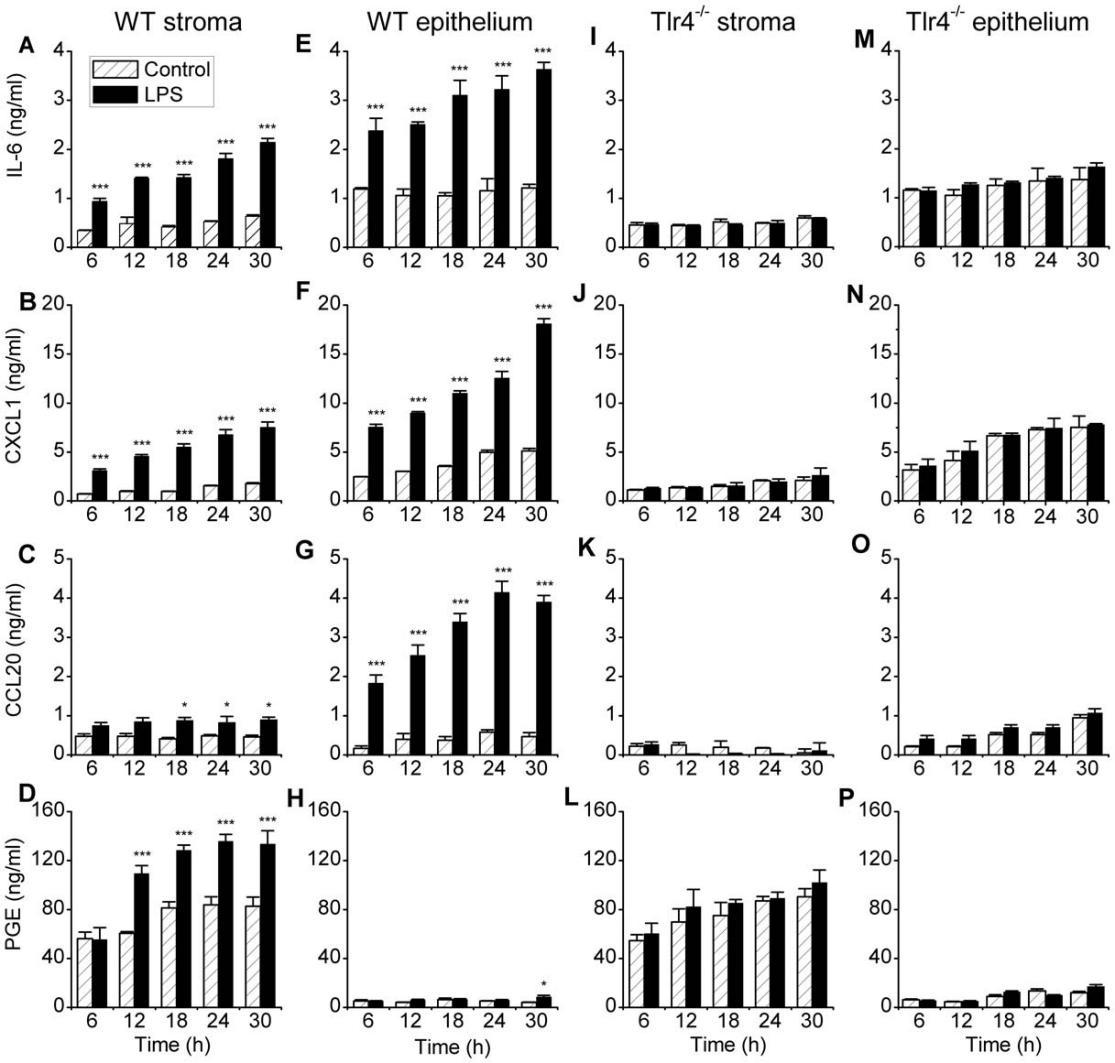
LPS stimulates secretion of inflammatory mediators by endometrial cells from wild type but not Tlr4^−/−^ mice. Endometrial stromal (A–D) and epithelial cells (E–H) isolated from WT mice, and stromal (I–L) and epithelial cells (M–P) isolated from Tlr4^−/−^ mice were cultured for 6, 12, 18, 24 or 30 h in control media (hatched open bars) or media containing 1 µg/ml ultrapure LPS from *E. coli* serotype O111:B4 (solid bars). Supernatants were harvested to measure the accumulation of IL-6 (A, E, I, M), CXCL1 (B, F, J, N), CCL20 (C, G, K, O), and prostaglandin E_2_ (PGE; D, H, L, P). LPS stimulated accumulation of IL-6, CXCL1, CCL20 and PGE in the supernatant of stromal and epithelial cells from WT mice but not Tlr4^−/−^ mice. Data are presented as mean + SEM and represent 3 independent experiments with 2 wells per treatment. Values differ between LPS-treatment and control media within time point when analysed using Student t-test, * P<0.05, *** P<0.001.

To explore the temporal response of endometrial cells isolated from WT mice, stromal and epithelial cells were cultured for 6 to 30 h in control media or media containing 1 µg/ml ultrapure LPS from *E. coli*. Supernatants of the stromal cells treated with LPS accumulated IL-6, CXCL1, CCL20 and PGE over time (ANOVA, P<0.001) and in a treatment dependent manner (ANOVA, P<0.001) for each inflammatory mediator. For LPS-treated stromal cells compared with stromal cells in control medium, increases in the concentration of the inflammatory mediators were evident by 6 h for IL-6 and CXCL1, and by 12 and 18 h for PGE and CCL20, respectively (Fig. 6A–D). Epithelial cell supernatants also accumulated more IL-6, CXCL1 and CCL20 in response to LPS over time (ANOVA, P<0.001) and in a treatment dependent manner (ANOVA, P<0.001) for each mediator. Epithelial cell IL-6, CXCL1 and CCL20 responses were evident following LPS treatment, compared with epithelial cells in control media, by 6 h (Fig. 6E–G) but an increase in PGE concentration in response to LPS was only detected at 30 h (Fig. 6H).

### TLR4 is necessary for the detection of LPS by endometrial cells *in vitro*

To examine the role of TLR4 in endometrial cells *in vitro*, stromal and epithelial cells were isolated from the endometrium of Tlr4^−/−^ mice [18]. These cells were cultured for 6 to 30 h in control media or media containing 1 µg/ml ultrapure LPS. The concentrations of the inflammatory mediators IL-6, CXCL1, CCL20 and PGE in the supernatant of stromal (Fig. 6I–L) or epithelial cells (Fig. 6M–P) did not differ significantly between control and LPS treated cells. As before, stromal or epithelial cell survival did not differ significantly between control and LPS treatment after 30 h (95.3±6.8 percent of control).

**Figure 7:**
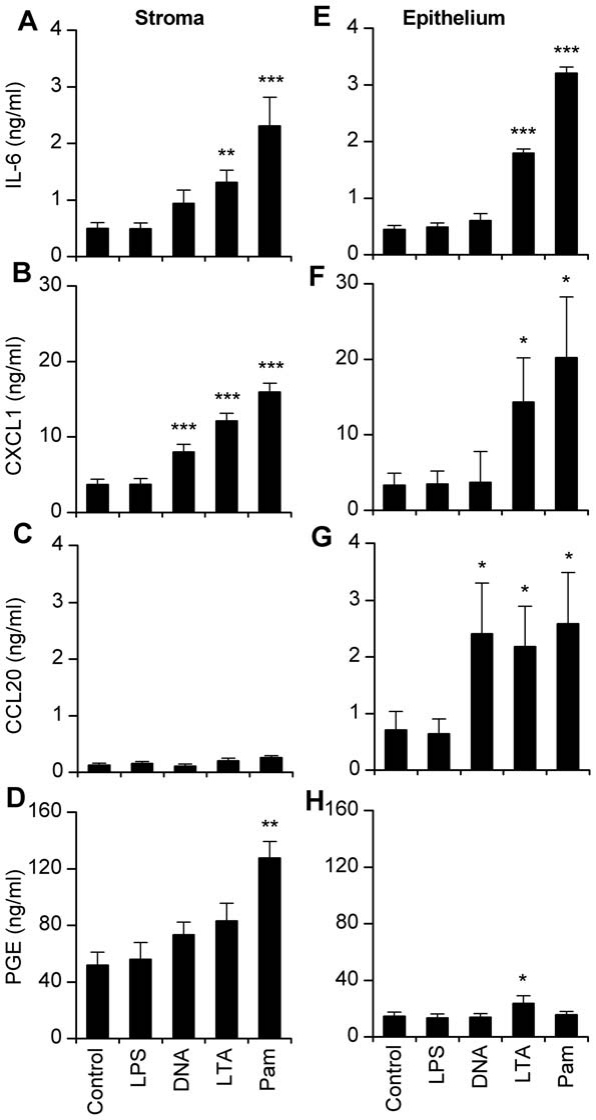
Endometrial cells from Tlr4^−/−^ mice respond to TLR ligands except LPS. Stromal (A–D) and epithelial cells (E–H) isolated from the endometrium of Tlr4^−/−^ mice, were cultured for 24 h in medium (Control), or media containing 1 µg/ml ultrapure LPS from *E. coli* O111:B4, 5 µg/ml bacterial DNA purified from *E. coli* (TLR9 ligand), 1 µg/ml purified *Staphylococcus aureus* lipoteichoic acid (LTA; TLR2 ligand), or 1 µg/ml synthetic tripalmitoylated lipopeptide Pam3CysSerLys4 (Pam; TLR1 and TLR2 ligand). Supernatants were harvested to measure the accumulation of IL-6 (A, E), CXCL1 (B, F), CCL20 (C, G), and prostaglandin E_2_ (PGE; D, H). Data are presented as mean + SEM and represent 3 independent experiments with 2 wells per treatment. Data were analysed by ANOVA, using the Dunnett's pairwise multiple comparison t-test to compare treatments with control, * P<0.05, ** P<0.01, *** P<0.001.

The lack of inflammatory response to LPS by the stromal and epithelial cells could be associated with a more widespread immune defect in the endometrial cells derived from Tlr4^−/−^ mice. So, to test if the endometrial cells from Tlr4^−/−^ mice were capable of innate immune responses to PAMPs acting though TLRs other than TLR4, the cells were treated for 24 h with: bacterial DNA purified from *E. coli* (TLR9 ligand), purified *Staphylococcus aureus* lipoteichoic acid (LTA; TLR2 ligand), or synthetic tripalmitoylated lipopeptide Pam3CysSerLys4 (Pam3CSK4; TLR1 and TLR2 ligand), as well as ultrapure LPS. The Tlr4^−/−^ stromal and epithelial cells did not respond to LPS, as expected, but did respond to the other PAMPs (Fig. 7A–H). In particular, stromal and epithelial cells responded to LTA and Pam3CSK4 with the accumulation of IL-6 (Fig. 7A, E) and CXCL1 (Fig. 7B, F). Cell survival was not significantly affected by treatment with PAMPs (99.1±1.8 percent of control).

### Stromal-epithelial cell interactions in the endometrial response to LPS

**Figure 8:**
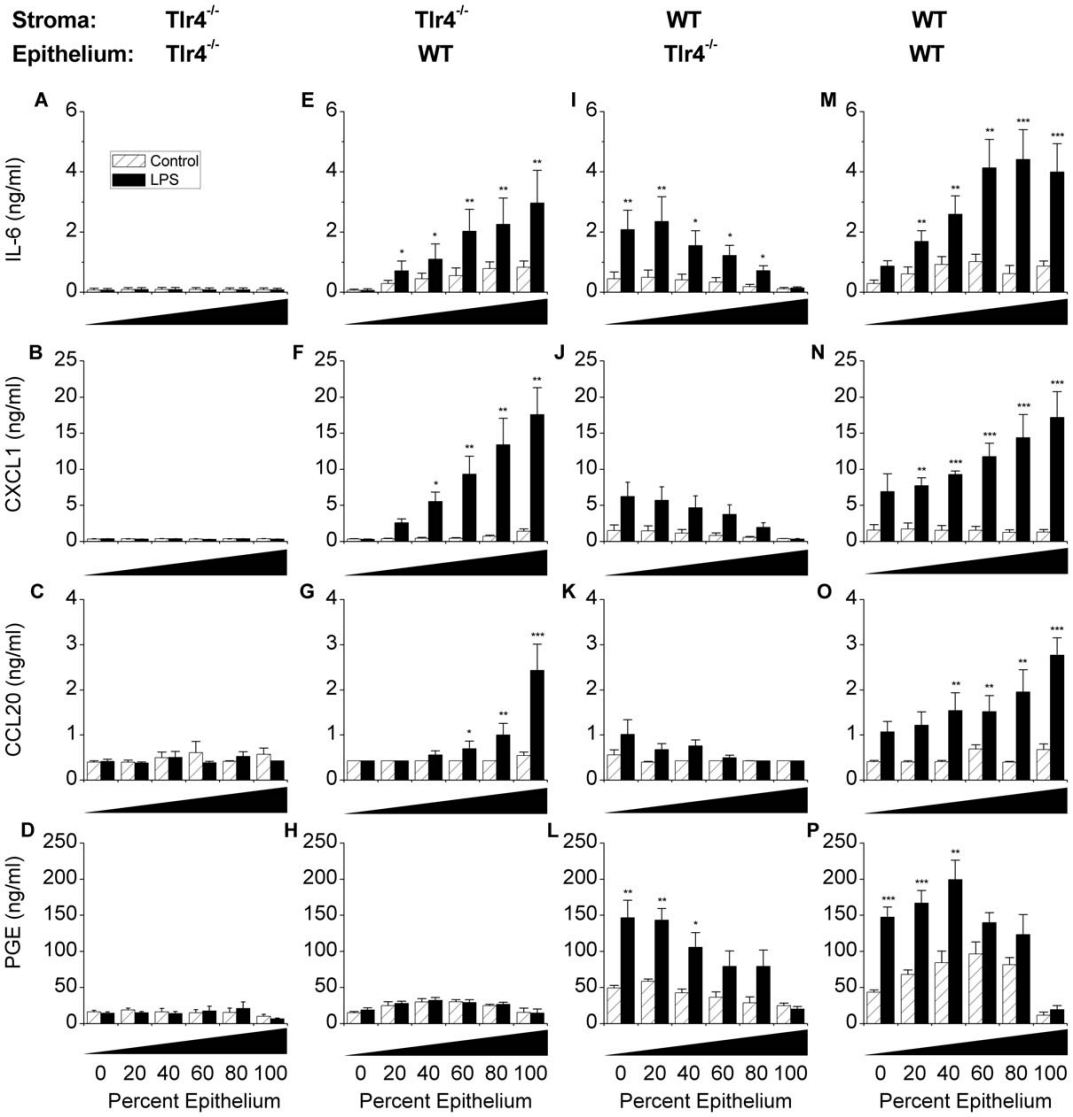
Stromal-epithelial interactions in the endometrium. Stromal and epithelial cells were isolated from Tlr4^−/−^ and wild type (WT) and co-cultured in 24-well plates with 0, 20, 40, 60, 80 or 100% epithelial cells per well. The combination of cells used was: Tlr4^−/−^ stroma with Tlr4^−/−^ epithelium (A–D), Tlr4^−/−^ stroma with WT epithelium (E–H), WT stroma with Tlr4^−/−^ epithelium (I–L), or WT stroma with WT epithelium (M–P). Cells were cultured for 24 h in control media (hatched open bars) or media containing 1 µg/ml ultrapure LPS from *E. coli* serotype O111:B4 (solid bars). Supernatants were harvested to measure the accumulation of IL-6 (A, E, I, M), CXCL1 (B, F, J, N), CCL20 (C, G, K, O), and prostaglandin E_2_ (PGE; D, H, L, P). LPS stimulated accumulation of IL-6, CXCL1, CCL20 and PGE in the supernatant of stromal and epithelial cells from WT mice but not Tlr4^−/−^ mice, principally in a cell type independent manner. Data are presented as mean + SEM and represent 3 independent experiments with 2 wells per treatment. Values differ between LPS-treatment and control media within co-culture group when analysed using Student t-test, * P<0.05, ** P<0.01, *** P<0.001.

In the female genital tract, stromal cells have a regulatory role for development, growth and differentiation of the overlying epithelium [20]. To explore if similar stromal-epithelial interactions had a role in innate immunity in the endometrium, co-cultures of stromal and epithelial cells were used containing final proportions of 0, 20, 40, 60, 80 or 100% epithelial cells. To examine the response to LPS, the stromal and epithelial cells derived from the endometrium of WT or Tlr4^−/−^ mice were used in four different combinations (Tlr4^−/−^ stroma with Tlr4^−/−^ epithelium, Tlr4^−/−^ stroma with WT epithelium, WT stroma with Tlr4^−/−^ epithelium, or WT stroma with WT epithelium), and treated with 1 ml of control media or media containing 1 µg/ml ultrapure LPS (Fig. 8). As expected, when stromal and epithelial cell were both derived from Tlr4^−/−^ mice, the concentrations of IL-6, CXCL1, CCL20 or PGE in supernatants did not differ significantly between control and LPS treated cells (Fig. 8A–D). The use of stromal cells from Tlr4^−/−^ mice with epithelial cells from WT mice (Fig. 8E–H), or *vice versa* (Fig. 8I–L), produced inflammatory mediator responses that were dependent on the proportion of WT epithelial cells or stromal cells, respectively. There was no evidence of interaction between the stromal and epithelial cells to increase or decrease the accumulation of inflammatory mediators. Similarly, when both stromal and epithelial cells were derived from WT animals there was no evidence of stromal-epithelial interactions for the production of IL-6, CXCL1 or CCL20 (Fig. 8M–O). The only evidence of an interaction between stromal and epithelial cells was enhanced accumulation of PGE by co-culture of stromal cells with 40% epithelial cells (Fig. 8P).

## Discussion

Pelvic inflammatory disease (PID), abortion and preterm labour are common diseases of women caused by bacterial infection of the upper female genital tract that causes pain, infertility and mortality [1], [3], [21]. Of course, inflammation is also a protective response by the body to counter detrimental stimuli and bacterial LPS is specifically detected by TLR4 to generate an innate immune response [9]. To examine the role of TLR4 in the detection and response to LPS in the endometrium, the present study used Tlr4^−/−^ mice [18]. Indeed, WT but not Tlr4^−/−^ mice infused intrauterine with LPS developed PID with influx of granulocytes into the endometrium. This was not a generalised response to LPS as there was little evidence of PID in mice infused intraperitoneal with LPS. Stromal and epithelial cells isolated from the endometrium of WT mice secreted inflammatory mediators when treated with LPS in a dose and time dependent manner. However, endometrial cells from Tlr4^−/−^ mice did not respond to LPS, although they were capable of an inflammatory response to other TLR ligands. These data support the concept that stromal and epithelial cells of the endometrium have important roles in innate immunity and are not bystanders during bacterial infection, at least during the initial stages.

The mouse has been used as a model to study PID and preterm labour by intrauterine infusion of bacteria or LPS [4], [5], [22]. However, bacteria have multiple PAMPS that stimulate several TLRs, and many LPS-infusion studies use standard preparations of lipopolysaccharide. These standard LPS preparations are extracted by a phenol-water mixture and contain other bacterial components, such as lipopeptides, and therefore stimulate TLR2 as well as TLR4. However, the ultrapure LPS used in the present study is extracted by successive enzymatic hydrolysis steps and purified by the phenol-triethylamine-deoxycholate extraction protocol [23]. This ultrapure LPS caused PID in WT but not Tlr4^−/−^ mice when infused intrauterine and the temporal accumulation of granulocytes in the endometrium of the WT mice was similar to the pathology of clinical disease [1], [4]. However, intraperitoneal injection of WT mice with LPS did not cause PID, supporting the concept that endometrial cells have a role in the localised detection and response to LPS in the female genital tract.

Endometrial cells of women, mice and cattle express the majority of TLRs at least at the level of mRNA [15], [24]–[27]. In the present study, immuno-reactive TLR4 protein was detected in the stromal and epithelial cell compartments, in the endometrium of WT mice. Furthermore, purified populations of endometrial cells from the WT mice secreted IL-6, CXCL1, CCL20 and PGE in response to LPS, in a dose and time dependent manner. The secretion of inflammatory mediators in response to LPS supports previous observations in murine endometrial epithelial cells [11], [15]. However, the present data highlight the importance of stromal cells during the inflammatory response to LPS. Although the epithelium is the first barrier to infection [28], the stromal cells are more abundant, so the stroma may make a greater contribution to inflammation once microbes or their PAMPs penetrate past the epithelium. The increased secretion of the chemokines CXCL1 and CCL20 is particularly important for attracting granulocytes to sites of infection [29]. The present data support previous observations that human endometrial cells secrete the chemokine IL-8 in response to LPS [14]. Epithelial cells from the mouse endometrium also secrete CCL20 constitutively and PAMPs rapidly stimulate accumulation of this chemokine in cell supernatants [11]. The secretion of PGE from stromal cells stimulated with LPS was not unexpected because PGE is an inflammatory mediator [10]. In addition, PGE has important roles in endometrial stromal but not epithelial cells, during decidualization and implantation [30]. There appeared to be less secretion of IL-1β and TNFα by endometrial cells in response to LPS than expected from work using macrophages [9], [15], [18]. This may reflect differences between endometrial cells and professional immune cells, such as an active inflammasome in macrophages where caspase-1 cleaves pro-IL-1β to its mature form [31].

The next step was to test if TLR4 was essential for the endometrial epithelial or stromal cell responses to LPS, using Tlr4 deficient mice [18]. Indeed, endometrial cells from Tlr4^−/−^ mice did not generate an inflammatory response to ultrapure LPS even after 30 h of treatment. This lack of response to LPS was not due to a wider defect in innate immunity because TLR1, TLR2 and TLR9 ligands stimulated secretion of IL-6, CXCL1, CCL20 and PGE from endometrial cells purified from Tlr4^−/−^ mice. There appeared to be differences between the Tlr4^−/−^ stromal and epithelial cells in their response to *Staphylococcus aureus* lipoteichoic acid and synthetic tripalmitoylated lipopeptide Pam3CysSerLys4, which may reflect underlying differences in the ability of the cells to recognize TLR2 ligands.

Stromal cells have a regulatory role for development, growth and differentiation of the overlying epithelium in the endometrium [20]. However, the co-culture of stromal cells from Tlr4^−/−^ mice with epithelial cells from WT mice, or *vice versa*, provided little evidence of interaction between the stromal and epithelial cells to modulate the accumulation of inflammatory mediators except for PGE, which may reflect the endocrine roles that PGE has in reproductive biology [30]. An interesting observation from the co-cultures was that the control responses tended to be higher when both cell types were from WT rather than Tlr4^−/−^ mice. This may represent increased innate immune activity of WT cells, perhaps mediated by detection of some damage associated molecular patterns generated by stressed cells *in vitro* [32], although cell survival was not grossly affected by LPS treatment for 24 h.

An unanswered question from the present study is whether TLR4 has any role in the reproductive biology of healthy organisms. However, this seems unlikely as infertility has not been reported for Tlr4^−/−^ mice. Another unanswered question is the role of immune cells in the endometrium. Whilst the present study supports the concept that the epithelial and stromal cells are, at least initially, important for the detection and response to LPS via TLR4, the possibility that resident immune cells in the endometrium play a key role *in vivo* cannot be discounted.

In summary, the innate immune receptor TLR4 was expressed in the endometrium of mice and TLR4 was essential for mice to develop PID when infused with LPS intrauterine. Similarly, TLR4 was essential for the inflammatory response to LPS by purified populations of epithelial and stromal cells *in vitro*. We conclude that epithelial and stromal cells of the endometrium have a key role in innate immunity, and TLR4 is essential for the detection of LPS in the endometrium.

## Materials and Methods

### Ethics statement

All animal procedures were conducted at the Royal Veterinary College Biological Services Unit under the UK Animal Scientific Procedures Act (1986), with the approval of the UK Government Home Office (Licence Number PPL 70/6424), Swansea University Ethical Review Panel and the Royal Veterinary College Local Ethical Review Committee.

### Infusion of LPS *in vivo*

Wild type (WT) C57BL/6 mice were purchased from Charles River Laboratories (Margate, Kent, UK) and Tlr4-deficient (Tlr4^−/−^) mice on the C57BL/6 genetic background were donated by Dr C. Bryant (University of Cambridge; originally provided by S. Akira, Osaka University, Japan). Breeding colonies were maintained under standardized conditions, in a pathogen-free environment, with access to water and standard rodent diet. Female WT or Tlr4^−/−^ mice 6–8 weeks old (n≥5 per treatment) were infused intrauterine or intraperitoneal with 100 µg ultrapure LPS from *E. coli* serotype O111:B4 (Invivogen, Toulouse, France) or vehicle; in each case the total volume was 100 µl. Intrauterine infusions were done using a 27 G needle inserted into the tip of the left uterine horn, which was visualised by a laparotomy incision. Intrauterine infusions were done under general anaesthesia, which was induced using a combination of 10 mg/ml xylazine and 90 mg/ml ketamine by intraperitoneal injection (total volume 0.15 to 0.25 ml), and maintained using ∼1.5% halothane in oxygen. The genital tract was collected 2, 4, 6, 12 or 24 h after intrauterine infusion and 24 h after intraperitoneal infusion.

### Histology

Each genital tract was fixed overnight in 10% formalin, transferred to 70% ethanol and paraffin embedded (FFPE). The FFPE sections were cut at 6 µm, deparaffinised, and stained using haematoxylin and eosin at the same time using a Leica Auto Stainer XL (Leica, Allendale, NJ, USA). Images of at least 4 sections per animal and at least 4 fields of view per section were examined by Mirax Scan and Mirax Viewer software (Zeiss, Jena, Germany). Sections were also immunostained using the following antibodies: CD11b (NB110-89474; Novus Biologicals Ltd., Cambridge, UK) to identify granulocytes, TLR4 (sc-10741; Santa Cruz Biotechnology Inc., Santa Cruz, CA, USA), pan cytokeratin (ab6401; Abcam Inc., Cambridge, MA, USA), and isotype sera (Jackson Immunoresearch Laboratories Inc., West Grove, PA, USA). Alexafluor 555 secondary detection antibodies (Invitrogen, Paisley, UK) were then used, with nuclei stained using DAPI in the mounting medium (Vector Labs, Burlingame, CA, USA). Four sections per animal and at least 4 fields per section were examined before representative images were collected using an epifluorescent microscope (Axio Imager.M1; Zeiss) and an AxioCam, with scale bars added using the software (Axiovision; Zeiss). The number of CD11b immuno-reactive cells were counted in 4 random areas averaged over 4 independent sections from each animal.

### Epithelial and stromal cell isolation, culture and treatment

Epithelial and stromal cells were isolated from the endometrium of uteri collected from WT or Tlr4^−/−^ mice, and cultured as described previously [33]. Briefly, uteri were removed and cut open lengthwise; 6 to 8 uteri were then pooled, and incubated with 0.25% trypsin (Invitrogen, Paisley, UK) and 2.5% pancreatin (Invitrogen) at 4°C for 60 min and at 22°C for a further 60 min. The uteri were transfered to 15 ml ice-cold Hanks Balanced Salt Solution (HBSS; Invitrogen) and vortexed to release epithelial cells, and this procedure was repeated four times. The resulting cell suspensions were pooled, 3 ml of fetal bovine serum (FBS, heat-treated, endotoxin-free; Biosera, Ringmer, East Sussex, UK) added, and centrifuged at 500× g for 7 min. The epithelial cells were resuspended in complete medium consisting of Dulbecco Modified Eagle Medium (DMEM, without phenol red)/Hams F-12 nutrient mixed 1∶1 (Sigma, Dorset, UK), supplemented with 10% FBS, 20 mM Hepes, 100 µg/ml streptomycin, 100 U/ml penicillin, and 2 mM l-glutamine (Sigma). After the removal of the epithelial cells, the pooled uteri were incubated for 30 min at 37°C in HBSS with 0.05% trypsin, 0.02% EDTA and 400 U/ml DNase (Invitrogen). After adding 2 ml FBS, the resulting cell suspension was filtered through a 20 µm mesh (Falcon, Oxford, UK) to remove debris, centrifuged at 500× g for 10 min, and the stromal cells resuspended in complete medium. Medium was replaced at 48 h intervals to remove non-adherent cells but the cells were not passaged, and cultures at 4 days contained <1% CD45 positive cells. Cell cultures were maintained at 37°C, 5% CO_2_ in air, in a humidified incubator. For experiments, 90% confluent cells 2 to 4 days after collection were disassociated using Accutase (Sigma), resuspended in complete medium, comprising of DMEM (without phenol red)/Hams F-12 nutrient mixed 1∶1 (Sigma), supplemented with 10% FBS, 20 mM Hepes, 100 µg/ml streptomycin, 100 U/ml penicillin, and 2 mM l-glutamine (all Sigma). The epithelial or stromal cell suspensions were then plated at a density of 5×10^5^ cells per well in 24-well plates (Helena Bioscience, Gateshead, UK) in 1 ml of complete medium and used when 90% confluent, usually 2 days later. For co-culture experiments, stromal and epithelial cells derived from the WT and TLR4^−/−^ mice were used in four combinations (Tlr4^−/−^ stroma with Tlr4^−/−^ epithelium, Tlr4^−/−^ stroma with WT epithelium, WT stroma with Tlr4^−/−^ epithelium, or WT stroma with WT epithelium), and the stromal and epithelial cell suspensions were mixed at a final density of 5×10^5^ cells/ml containing 0, 20, 40, 60, 80 or 100% epithelial cells. These cell suspensions were plated using 1 ml/well in 24-well plates (Helena Bioscience) and treated when confluent, usually 2 days later. Experiments were repeated on three separate occasions with each treatment applied in duplicate.

Cells were treated in 24-well plates with the following PAMPs: ultrapure LPS from *E. coli* serotype O111:B4 (Invivogen); LPS purified from *E. coli* O55:B5 (Sigma); LPS purified from endometrial pathogenic *E. coli* serotype O73:H16 isolated from the endometrium of cattle with PID [4]; bacterial DNA purified from *E. coli* (Invivogen); purified *Staphylococcus aureus* lipoteichoic acid (LTA; Invivogen); synthetic tripalmitoylated lipopeptide Pam3CysSerLys4 (Pam3CSK4; Invivogen). The concentrations of PAMPs used to treat the cells were those suggested by the manufacturer (Invivogen): 1 µg/ml LPS, 5 µg/ml bacterial DNA, 1 µg/ml purified LTA, and 1 µg/ml Pam3CysSerLys4; except for the LPS does response experiments when cells were treated with a range of concentrations of ultrapure LPS from *E. coli* serotype O111:B4 (Invivogen) using a ten-fold increasing scale (1, 10, 100, 1000, or 10,000 ng/ml LPS). Experiments were performed using 1 ml of treatment medium per well in 24-well plates. Co-cultures were incubated with 1 ml control medium or 1 ml medium containing 1 µg/ml ultrapure LPS from *E. coli* serotype O111:B4 (Invivogen). Supernatants were collected 24 h after treatment or for time course experiments 6, 12, 18, 24 and 30 h after treatment, as indicated in *Results*, and stored at −20°C until required for measurement of the concentration of inflammatory mediators. Cell survival was assessed colorimetrically by the mitochondria-dependent reduction of MTT (Sigma) to formazan, as previously described [34].

### Inflammatory mediators

The concentrations of IL-1β, IL-6, TNFα, CXCL1, and CCL20 were measured by ELISA according to the manufacturer's instructions (DuoSet® ELISA Development Systems, R&D Systems, Inc., Minneapolis, MN, USA). The concentrations of PGE were measured by radio-immunoassay (RIA) as previously described [17]. Briefly, samples were diluted in 0.05 M Tris buffer containing 0.1% gelatin and 0.01% sodium azide. Standards and tritiated tracers were purchased from Sigma and Amersham International PLC (Amersham, UK), respectively. The antisera were a generous gift from Professor N.L. Poyser (University of Edinburgh, UK) and their cross-reactivity have been reported [35]. The limit of detection for PGE was 2 pg/tube and intra- and inter-assay coefficients of variation were 4.4% and 7.8%, respectively.

### Statistical analysis

Data analysis was performed using SPSS ver 13.0 (SPSS Inc., Chicago, USA). Data were analysed using ANOVA, using the Dunnett's pairwise multiple comparison t test to compare treatments with control. Student's t-test was used to compare differences between 2 independent samples. Non-parametric data were examined using Mann-Whitney *U* or Kruskal-Wallis tests for 2 or more independent samples, respectively. Results are quoted as mean ± SEM, and significance attributed when P<0.05.
